# Effect of Supplementation with Red Rooibos Tea on Body Composition, Metabolic Outcomes, and Movement in Ovariectomized Sprague-Dawley Rats

**DOI:** 10.1016/j.cdnut.2026.107656

**Published:** 2026-02-06

**Authors:** Rebekah S Feld, Mitchell A Ianiero, Matthew K Goncharow, Dawson B Kays, Sara Madanat, Rebecca EK MacPherson, Brian D Roy, Wendy E Ward

**Affiliations:** 1 Department of Kinesiology, Brock University, St. Catharines, ON, Canada; 2 Department of Health Sciences, Brock University, St. Catharines, ON, Canada; 3 Centre for Bone and Muscle Health, Faculty of Applied Health Sciences, Brock University, St. Catharines, ON, Canada

**Keywords:** ovariectomy, body composition, menopause, rats, polyphenols

## Abstract

**Background:**

Menopause is associated with increases in visceral adipose tissue, reductions in lean mass, and a reduced energy expenditure. The ovariectomized (OVX) rat model can be used to model these changes and to test potential interventions that may attenuate this response. Red rooibos (RR), due to its high polyphenol content, may counter the effect of low estrogen concentrations.

**Objectives:**

To determine if consumption of RR can blunt the changes to body composition, metabolic outcomes, and movement that occur in the OVX rat model.

**Methods:**

Fifty-six female, 6-mo-old, Sprague-Dawley rats were randomly assigned to 4 groups (*n* = 14 per group): SHAM-WATER (sham surgery and consumed water without RR), SHAM-RR, OVX-WATER, and OVX-RR. RR (2.6 g RR per kilogram body mass) was provided *ad libitum* for 12 wk. At baseline and endpoint, body composition was measured using dual energy X-ray absorptiometry, and metabolic measures and movement were measured using a Promethion metabolic caging system.

**Results:**

RR did not attenuate OVX-induced changes. As expected, OVX resulted in a higher whole-body percent fat mass, a lower percent lean mass, and a higher body mass than SHAM rats (*P* < 0.05). At the endpoint, OVX rats had a higher ovarian adipose tissue mass and inguinal adipose tissue mass (*P* < 0.05) with a trend for reduced brown adipose tissue mass than SHAM rats (*P* = 0.072). There were no differences between groups for metabolic measures (energy expenditure, gas exchange). OVX rats also had a trend of a long lounge time (*P* = 0.065) and total lounge time (*P* = 0.070) compared with SHAM.

**Conclusions:**

RR intervention did not attenuate the changes to body composition, metabolic, or movement outcomes observed in the OVX group. This study provides insights into the progression of body composition and metabolic changes in the OVX rat model, contributing to a better understanding of the physiological impacts of estrogen deficiency.

**Statement of significance**

Provides comprehensive metabolic phenotyping including body composition with accompanying *in vivo* metabolic measures such as energy expenditure and movement in the rat ovariectomy model.

## Introduction

Natural menopause is defined as a lack of menstrual cycle, or amenorrhea, for 12 mo in females aged ≥45 y [1]. Over 10 million females in Canada are over the age of 40 y, representing around 1 quarter of the population [2]. Menopause is widely recognized to impact body composition and body fat distribution, with estrogen loss driving increases in visceral adipose tissue [[3], [4], [5]]. Studies have shown that ovariectomized (OVX) rodents provided with exogenous estrogen have decreased food intake and increased energy expenditure (EE) and thus are protected against the accumulation of fat mass [6,7].

Green tea—whole or as an extract has been shown to attenuate fat mass gain in rats fed a high fat diet (HFD) [8,9]. Moreover, green tea polyphenol supplementation in HFD-induced obese rats has been shown to reduce markers of inflammation, decrease percent fat mass, and increase percent fat-free mass [8]. In humans, several studies have shown that providing green tea polyphenols, particularly epigallocatechin-3-gallate, increases EE and fat oxidation [[10], [11], [12], [13]]. Green tea may also have estrogenic activity [[14], [15], [16]]. In vitro studies have shown that green tea may increase osteogenesis and inhibit adipocyte formation in bone marrow-derived mesenchymal cells, which represent an important link to adipose tissue in postmenopausal females [17].

Whether other teas, such as red rooibos (RR), have a similar effect via estrogenic and/or antioxidant activity and may thereby attenuate the effects of OVX is unknown. The polyphenol profile of RR differs from that of green tea, as they come from different plants—RR is from the *Aspalathus linearis* plant, whereas green tea is from the *Camellia sinensis* plant [18]. The dominant polyphenols found in green tea are catechins, of which epigallocatechin-3-gallate is the most abundant, followed by (‒)-epicatechin, (‒)-epigallocatechin gallate, (‒)-epigallocatechin, (+)-catechin, and (+)-gallocatechin [19]. Conversely, the dominant polyphenol profile of RR tea includes the dihydrochalcone aspalathin, along with flavones orientin, isoorientin, luteolin, and rutin [20]. However, due to the similar structure among polyphenols in teas, the beneficial effects of green tea and its extracts demonstrated in preclinical and human studies provide a basis for studying the effects of supplementation with RR. Moreover, unlike green tea, RR does not contain caffeine, and thus, providing RR at supplemental amounts removes concern regarding exposure to caffeine.

The OVX rat model is considered the gold standard in mimicking the physiological effects of estrogen loss that occur with menopause in humans [21]. For example, significant increases in body mass and changes to body composition after OVX have been consistently reported [[22], [23], [24], [25], [26]]. However, the metabolic effects of RR have not previously been examined in the OVX model or in the context of estrogen deficiency. Thus, this study addressed this research gap with an objective to investigate whether an RR intervention could attenuate the changes in body composition (fat mass and lean mass) that occur post-OVX and to also determine the corresponding changes in metabolic (EE, gas exchange) and movement outcomes.

## Methods

### Experimental model and design

Fifty-six female Sprague-Dawley rats (3 mo old, 14 rats per group) were purchased from Charles River Laboratories and acclimatized at the Brock University Animal Facility for 1 wk. To accommodate the number of metabolic cages available, rats were ordered in 4 batches, staggered by 2 wk (batches 1‒3: *n* = 16; batch 4: *n* = 8). Within each batch, rats were stratified by body mass such that each pair of rats in a cage had a similar body mass, and cages were randomly assigned to 1 of 4 intervention groups (SHAM + water; OVX + water; SHAM + RR; or OVX + RR; *n* = 14 per group). SHAM + RR had a final size of *n* = 13 due to a pre-surgical euthanasia following veterinary consultation. The remaining rat was housed with 2 others within the same intervention group. From arrival to 6 mo old, rats had free access to both reverse osmosis (RO) water and Teklad Global 14% protein maintenance diet (Inotiv), *ad libitum*. At 6 mo of age, all rats underwent their assigned procedures, either a bilateral OVX or SHAM surgery. During the OVX surgery, both ovaries were surgically removed to induce a rapid decline in estrogen production. The SHAM procedure was identical to the OVX, excluding the removal of the ovaries. All surgeries were completed in-house within the Brock Animal Facility by certified animal care technicians (KB and SB). Following the procedures, all rats were provided an AIN-93M diet (Inotiv) and consumed RO water, or RR prepared using the same water.

Water or RR intake was measured and recorded 3 times per week, whereas food intake and body mass were measured and recorded once weekly. All rats were pair-housed in a 12:12 light-dark cycle with a temperature of 20°C. Environmental enrichment included double-decker housing, allowing the rats to climb, jump, and stand (Tecniplast), along with a plastic tunnel and crinkle nest. This study was carried out in adherence to the Canadian Council on Animal Care guidelines and was approved by the Brock Animal Care Committee (AUP 23-02-01).

### Preparation of RR tea

RR was provided by Cape Natural Tea Products. RR was put into paper bags and steeped in boiling RO water (96°C) for a total of 6 min as previously described [27]. The tea was left to cool for 15 min prior to being divided into bottles. To minimize the degradation of polyphenols, tea was placed in UV-resistant bottles (Tecniplast) and prepared fresh every 2‒3 d. A parallel study [28] investigated the changes in polyphenol activity over time using the same RR used in the present study. The inhibition of free radical 2,2-diphenyl-1-picrylhydrazyl was used to determine polyphenol antioxidant activity over 6 successive days at 24-h intervals. Polyphenol antioxidant activity was affected by 2‒3 d after steeping.

RR was provided at an amount of 2.6 g RR tea per kilogram body mass per day, as per a previous study that had calculated the amount of RR to be equivalent to human consumption of ∼12 cups/d and thus realistically would be consumed as a supplement and not through direct consumption of RR tea [27]. Throughout the 12-wk intervention, RR tea was prepared separately for each cage, using the mean of the weekly body mass for the 2 rats in the cage. To help ensure a similar body mass between the 2 rats, rats had been stratified by body mass at time of randomization to the various groups.

### Body composition using dual energy X-ray absorptiometry

Rats were selected in a random order to complete body composition scans at baseline and endpoint using the iNSiGHT VET dual energy X-ray absorptiometry (OsteoSys). Rats were anesthetized with 3.2% isoflurane gas for 5 min until they relaxed and were asleep. Anesthesia was then immediately removed as the rats remained still during the scan. Rats were placed in a prone position; legs and arms outstretched with the tail curved under the left foot, and the scan was completed. High-resolution images were obtained using a cone beam X-ray source that generated low (60 kV/0.8 mA) and high (80 kV/0.8 mA) energy X-rays. Each scan was completed in ∼20 s. The fat and lean mass of the whole body and a region of interest (ROI) were measured. The ROI encompassed the visceral region via placement of a rectangle that aligned with the top of L2, whereas the bottom of the rectangle aligned with the lower edge of L5. Insight software (version 1.0.6; OsteoSys) was used to analyze images. Fat and lean mass were calculated as a percent of total body mass and as an absolute mass for the whole body and the ROI.

### Metabolic (EE, gas exchange) and movement outcomes using a metabolic cage system

The Promethion Core rodent metabolic cage system was used at baseline and the study endpoint (Sable Systems International) as previously reported [29]. Within each batch of rats, cages from the 4 different groups were selected in random order for placement in the metabolic cage system. Rats were individually housed in these cages on an open shelving unit in a temperature, light, and noise-controlled environment for 48 h. There was a 24-h adjustment period followed by a 24-h period in which data were collected using the metabolic cage system. This included food and water or RR intake, mean oxygen consumption (VO_2_), EE (calculated by system using the Weir equation), respiratory exchange ratio [RER; as calculated by carbon dioxide production (VCO_2_)/VO_2_], as well as movement patterns including direct and indirect locomotion, mean ambulatory speed, fine motor activity, sedentary time, sleep time, and other activities related to sleeping, feeding and drinking. Previous work has established that lean mass is a determinant of EE and contributes to ∼50%‒80% of basal EE [30]. For this reason, the EE and VO_2_ measurements were normalized to lean mass to ensure results were not influenced by the changes in lean mass that occurred over time [19,31]. Time budget data was extrapolated by creating a daily time budget for each cage, presented as a percentage of total cycle time spent eating food, touching the food hopper, drinking water/tea, touching the drink hopper, time within the “home” structure, touching the “home,” long lounge time, and short lounge time. Data was recorded and compiled using the Promethion Live software system (Sable Systems).

### Peri-ovarian adipose tissue, inguinal white adipose tissue, brown adipose tissue, and uterine mass

At the study endpoint, rats were anesthetized via 5% isoflurane gas administration and then euthanized via exsanguination through cardiac puncture. Adipose tissue from 3 sites [peri-ovarian adipose tissue (POAT), inguinal white adipose tissue (iWAT), and brown adipose tissue (BAT)] were excised and weighed. Adipose tissue mass was standardized to body mass to calculate the ratio of adipose tissue mass to body mass. Uterine mass was measured to confirm the success of the OVX surgery.

### Statistical analyses

The current study is part of a larger study that has a primary outcome of bone structure, specifically the ratio of bone volume to total volume measured by micro-computed tomography. The required sample size, based on that outcome, was determined to be 12 per group based on a previous study [27]. An additional 2 rats per group were included to safeguard against unforeseen losses. Statistical analyses were conducted using SPSS Statistics (version 27, IBM). The effect of surgery (SHAM and OVX), intervention (WATER and RR), and the interaction on food and water intake, body mass, body composition (fat and lean mass: absolute and percentage for each ROI), and metabolic cage data (VO_2_, EE, RER, direct and indirect locomotion, mean ambulatory speed, fine motor activity, sedentary time, and sleep time) were evaluated through a mixed analysis of variance with repeated measures. Within-subjects analyses were performed between baseline and endpoint data, whereas between-subjects analyses were performed between groups (SHAM-WATER, SHAM-RR, OVX-WATER, OVX-RR). Any single time point measures (i.e., adipose tissue mass at various sites) were analyzed using a 2 × 2 analysis of variance (intervention × surgery). Differences between means were deemed significantly different if *P* < 0.05. When a significant interaction was identified, a Bonferroni post hoc was performed to test the main effects at each time point. Results with significance (0.05 < *P* < 0.10) were noted and interpreted cautiously. If data were unavailable (e.g., technical issues with the dual energy X-ray absorptiometry or metabolic caging, animal loss), the sample size is noted in the corresponding figure or table. Remaining data for the rat were included for other outcomes (weekly measures, adipose tissue mass, and data from housing in a metabolic cage).

## Results

### Supplementation with RR did not blunt OVX-induced changes in food intake, fluid intake, or body mass

**FIGURE 1:**
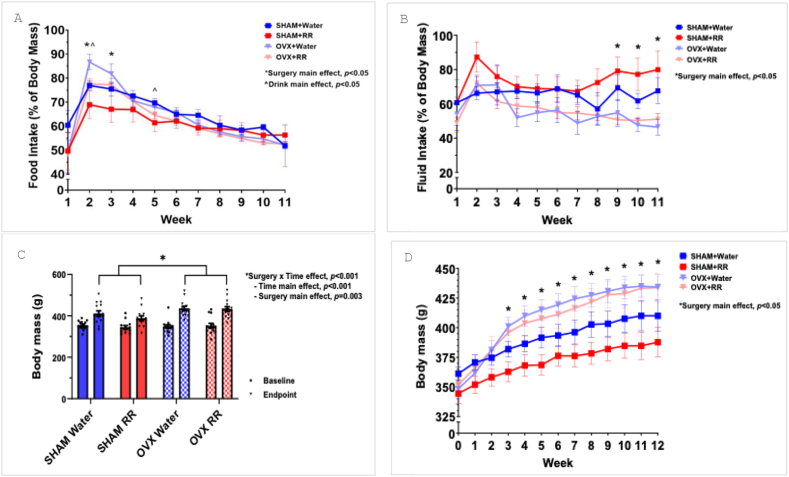
Food and fluid intake and body mass. (A) Weekly food intake as a percentage of body mass. (B) Weekly fluid intake as a percentage of body mass. (C) Body mass at baseline (week 0) and endpoint (week 12). (D) Weekly body mass throughout the 12-wk intervention. *n* = 14 in all groups except for SHAM-RR, which had *n* =13. OVX, ovariectomy; RR, red rooibos.

RR tea had no effect on food intake, though OVX rats consumed a greater amount of food over time than SHAM rats (*P* < 0.05). Weekly food intake was higher (*P* < 0.05) in OVX rats at weeks 2 and 3, and groups receiving water without RR consumed more food (*P* < 0.05) than RR groups at weeks 2 and 5 (Figure 1A). RR had no effect on fluid intake, whereas SHAM rats consumed a greater quantity of fluid, regardless of whether they received water or RR, at weeks 9 through 11 (*P* < 0.05). Similarly, overall fluid intake was different between surgery groups, where SHAM rats consumed more fluid overall (*P* = 0.024) than OVX rats (Figure 1B).

There were no differences in body mass between groups at baseline (*P* = 0.866) (Figure 1C and D). At the endpoint, RR did not affect body mass, though OVX rats had a higher (*P* = 0.003) body mass than SHAM rats (Figure 1C and D). This difference began at week 3 of the study (*P* < 0.05) and continued through to the endpoint (Figure 1D)

### RR supplementation did not attenuate OVX-induced alterations in body composition

#### Whole body

**FIGURE 2:**
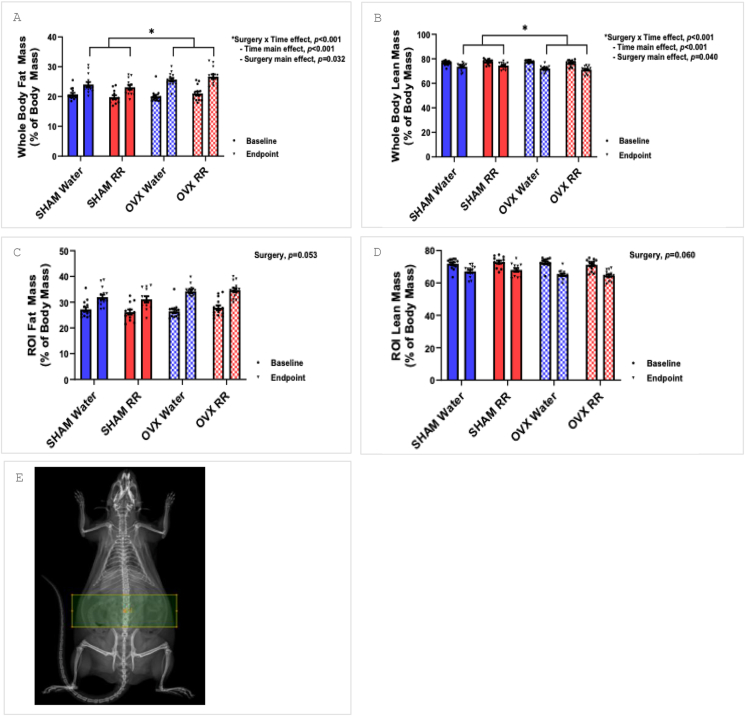
Body composition. Whole-body (A) fat mass and (B) lean mass, and ROI (C) fat mass and (D) lean mass at baseline and endpoint. (E) ROI as highlighted by the green box. However, *n* =14 in all groups except for SHAM-RR, which had *n* = 12. OVX, ovariectomy; ROI, region of interest; RR, red rooibos.

There was no effect of the RR intervention on body fat or lean mass. A time × surgery interaction was observed for whole-body percent fat mass and lean mass (*P* < 0.001; Figure 2A and B). Post hoc analyses revealed that although all rats experienced an increase in percent fat mass and a reduction in percent lean mass over time (*P* < 0.001), OVX rats experienced a greater increase in percent fat mass than SHAM, resulting in a higher whole-body percent fat mass and lower percent lean mass in OVX at endpoint (*P* = 0.032, *P* = 0.040; Figure 2A and B; Supplementary Table 1). All rats gained absolute whole-body fat mass and lean mass over time (*P* < 0.001). A time × surgery interaction was found for absolute fat mass and lean mass (*P* < 0.001). Post hoc analyses revealed OVX rats had higher absolute whole-body fat mass than SHAM at endpoint (*P* = 0.035), but no difference in absolute lean mass was found between the groups (Figure 2A and B, Supplementary Table 1).

#### ROI

There was no effect of RR, surgery, or time on percent fat or lean mass, or absolute fat or lean mass in the ROI. OVX rats trended to have a heavier percent fat mass (*P* = 0.053) and absolute fat mass (*P* = 0.065) compared with SHAM rats and a smaller percent lean mass (*P* = 0.060) compared with SHAM rats (Figure 2C‒E; Supplementary Table 1).

### OVX resulted in higher POAT, iWAT, and lower BAT with no effect of RR supplementation

**FIGURE 3:**
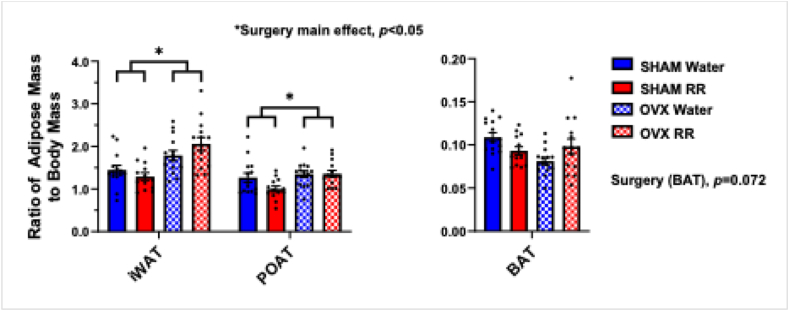
Adipose tissue mass. Ratio of iWAT, POAT, and BAT mass to body mass. However, *n* = 14 in all groups except for SHAM-RR, which had *n* = 13. BAT, brown adipose tissue; iWAT, inguinal white adipose tissue; OVX, ovariectomy; POAT, peri-ovarian adipose tissue; RR, red rooibos.

There was no effect of RR on adipose tissue mass at any site measured. The ratio of POAT and iWAT to body mass was both significantly greater in OVX compared with SHAM (*P* = 0.012; *P* < 0.001) groups. A surgery × intervention interaction was found for the ratio of BAT to body mass, and post hoc analysis revealed, within the intervention group, a higher ratio in the SHAM than in the OVX (*P* = 0.002) groups. There was a trend of a lower ratio of BAT to body mass in OVX compared with SHAM (*P* = 0.072) groups (Figure 3). Uterine mass was heavier in SHAM (0.65 ± 0.02 g) compared with OVX (0.15 ± 0.02 g) (*P* < 0.001) groups, confirming the success of the OVX procedure.

### Supplementation with RR did not alter metabolic measures or movement data

#### EE

There was a surgery × RR intervention x time interaction for 24-h EE (*P* = 0.044). Post hoc analyses revealed that all rats had a higher mean 24-h EE at endpoint than at baseline (*P* = 0.019), whereas RR intervention and surgery were not significant (Table 1).

**TABLE 1:** Summary of oxygen consumption, respiratory exchange ratio, and energy expenditure measurements >24-h or a 12-h light or dark cycle

		SHAM		OVX		*P* values						
		water	RR	water	RR	Surgery	Intervention	Time	Surgery × intervention	Surgery × time	Intervention × time	Surgery × intervention × time
		*n* = 14	*n* = 12‒13	*n* = 14	*n* = 14							
Measures^1^												
Av.VO_2_ 24-h/LM	1	1574 ± 30.8	1519 ± 37.0	1579 ± 35.6	1592 ± 36.5	NS	NS	NS	NS	0.039^2^	0.022^4^	NS
(mL/kg LM/h)	2	1554 ± 54.6	1667 ± 49.7	1534 ± 42.5	1583 ± 45.2							
Av.VO_2_ light/LM	1	1396 ± 41.2	1353 ± 31.6	1414 ± 38.0	1395 ± 29.4	NS	NS	0.004	NS	0.021^2^	0.034^4^	NS
(mL/kg LM/h)	2	1428 ± 55.5	1531 ± 42.6	1416 ± 41.9	1419 ± 41.8							
Av.VO_2_ dark/LM	1	1750 ± 29.0	1675 ± 46.9	1744 ± 40.2	1779 ± 50.5	NS	NS	NS	NS	NS	0.022^3^	NS
(mL/kg LM/h)	2	1681 ± 57.4	1803 ± 60.7	1652 ± 50.4	1747 ± 51.8							
Av. RER 24-h	1	0.99 ± 0.01	0.98 ± 0.01	0.96 ± 0.01	0.96 ± 0.01	NS	NS	NS	NS	NS	NS	NS
	2	0.96 ± 0.02	0.99 ± 0.01	0.98 ± 0.02	0.95 ± 0.02							
Av. EE 24-h/LM	1	0.132 ± 0.003	0.126 ± 0.003	0.132 ± 0.003	0.131 ± 0.003	NS	NS	0.019	NS	NS	0.010^4^	0.044^5^
(kcal/kg LM/min)	2	0.130 ± 0.005	0.140 ± 0.004	0.134 ± 0.004	0.135 ± 0.004							
Av. EE light/LM	1	0.117 ± 0.003	0.112 ± 0.003	0.117 ± 0.005	0.114 ± 0.003	NS	NS	NS	NS	NS	0.032^4^	0.042^5^
(kcal/kg LM/min)	2	0.118 ± 0.005	0.128 ± 0.004	0.123 ± 0.004	0.120 ± 0.004							
Av. EE dark/LM	1	0.147 ± 0.002	0.140 ± 0.004	0.147 ± 0.004	0.147 ± 0.004	NS	NS	NS	NS	NS	0.007^4^	NS (0.096)
(kcal/kg LM/min)	2	0.141 ± 0.005	0.152 ± 0.005	0.145 ± 0.005	0.149 ± 0.004							

Abbreviations: Av., mean; EE, energy expenditure; LM, lean mass; NS, not significant; OVX, ovariectomy; RER, respiratory exchange ratio; RR, red rooibos; VO2, oxygen consumption.

0.05 < *P* < 0.10 is denoted as NS (*P*) to display trends.

Timepoint 1 represents baseline data. Timepoint 2 represents endpoint data.

1 Values are mean ± SEM (*P* > 0.05).

2 A significant difference exists between SHAM at baseline and SHAM at endpoint.

3 A significant difference exists between Water at baseline and Water at endpoint.

4 A significant difference exists between RR at baseline and RR at endpoint.

5 A significant difference exists between SHAM-RR at baseline and SHAM-RR at endpoint.

#### Gas exchange

There were interactions for surgery × time and RR intervention × time for mean 24-h VO_2_ (*P* = 0.039; *P* = 0.022) and mean light cycle (12-h) VO_2_ (*P* = 0.021; *P* = 0.034) when standardized to lean mass (Table 1). Post hoc analyses revealed an effect of time in mean light cycle VO_2_ (*P* = 0.004), representing an overall increase in mean light cycle VO_2_ over time. All other post hoc findings were nonsignificant. There was no significant effect of time, RR intervention, or surgery for RER in 12-h dark or light cycle, or 24-h periods (Table 1).

#### Movement and time budget data

**FIGURE 4:**
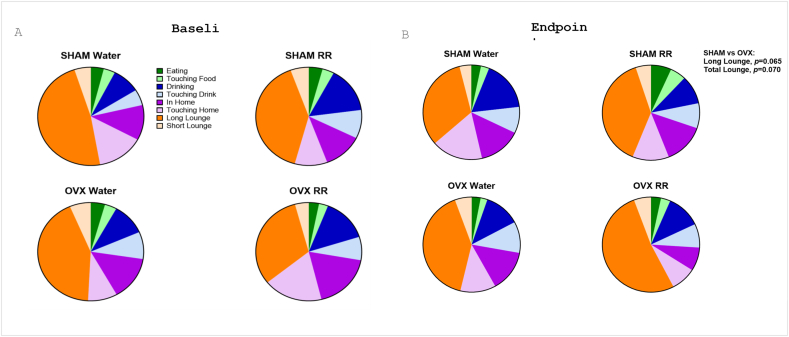
Time budget data. (A) baseline and (B) endpoint. All measurements were performed using the Promethion metabolic cage system. However, *n* = 14 in all groups except for SHAM-RR, which had *n* = 13. OVX, ovariectomy; RR, red rooibos.

There was a trend in difference between surgery groups within mean still time (*P* = 0.085) and mean sleep time (still >40 s; *P* = 0.063), as a percentage of movement over a 24-h period (Table 2). Mean pedometer speed (centimeters per second), pedometer meters, and cage ambulation (meters) had no significant differences between intervention or surgery groups in the 12- or 24-h periods (Table 2). No significant differences were found among groups for any of the time budget activities measured (eating, touching food, drinking, touching drink, in-home, touching home, long lounge, and short lounge) at baseline or endpoint (Figure 4). However, trends were found for long lounge time (*P* = 0.065) and total lounge time (short lounge + long lounge; *P* = 0.070), where OVX rats had longer lounge time compared with SHAM rats at endpoint (Figure 4B). Within the OVX group, there was a significant surgery × time interaction in which mean fine motor activity was reduced over time (*P* = 0.012).

**TABLE 2:** Summary of movement data >24-h period

		SHAM		OVX		*P* values						
		water	RR	Water	RR	Surgery	Intervention	Time	Surgery × intervention	Surgery × time	Intervention × time	Surgery × intervention × time
		*n* = 14	*n* = 13	*n* = 14	*n* = 14							
**Measures**^1^												
Av. ped speed	1	1.38 ± 0.08	1.33 ± 0.05	1.40 ± 0.06	1.33 ± 0.06	NS	NS	0.046	NS	NS	NS	NS
Total (cm/sec)	2	1.42 ± 0.11	1.47 ± 0.07	1.64 ± 0.11	1.37 ± 0.10							
Av. ped meters	1	296 ± 31.1	322 ± 27.0	323 ± 21.1	296 ± 33.9	NS	NS	NS	NS	NS	NS	NS
Total (m)	2	311 ± 29.8	332 ± 28.4	341 ± 31.8	282 ± 33.4							
Av. cage	1	343 ± 31.3	366 ± 24.9	374 ± 20.6	357 ± 31.2	NS	NS	NS	NS	NS	NS	NS
Ambulation total	2	361 ± 30.3	378 ± 29.2	380 ± 31.5	326 ± 34.2							
Av. fine motor	1	46.3 ± 2.71	41.3 ± 5.53	51.0 ± 2.40	45.3 ± 3.62	NS	NS	NS	NS	0.012^2^	NS	NS
activity total (m)	2	49.6 ± 2.90	45.9 ± 2.91	39.3 ± 3.42	42.6 ± 2.66							
Av. still (%)	1	63.9 ± 1.74	56.4 ± 2.64	56.3 ± 2.72	57.8 ± 2.87	NS	NS	NS	NS	NS (0.085)	NS	NS
Total	2	56.8 ± 2.94	57.9 ± 3.25	59.9 ± 3.14	60.0 ± 3.23							
Mean sleep (%)	1	46.8 ± 1.76	36.6 ± 2.92	38.7 ± 2.97	39.6 ± 3.08	NS	NS	NS	NS	NS (0.063)	NS	NS
Total	2	38.8 ± 2.73	38.3 ± 3.43	42.4 ± 3.29	42.7 ± 3.37							

Abbreviation: Av., mean; NS, not significant; OVX, ovariectomy; RR, red rooibos.

0.05 < *P* < 0.10 is denoted as NS (*P*) to display trends.

Timepoint 1 represents baseline data. Timepoint 2 represents endpoint data.

1 Values are mean ± SEM (*P* > 0.05).

2 A significant difference exists between OVX at baseline and OVX at endpoint.

## Discussion

This study showed that RR supplementation did not attenuate the changes to body composition, EE, and movement that are induced by OVX in a rat model. Although the lack of effect of RR on these outcomes in the OVX model is a novel finding—particularly due to the inclusion of body composition and measures of EE and 24-h movement using metabolic caging—the study findings also add to the body of literature about the OVX rat used to model changes that occur in postmenopausal females.

Of note is that the dose of RR tea was at a supplemental amount and approximated the consumption of 12 cups of RR tea per day in humans, such that it is unlikely that a higher dose of RR would modulate the effect of OVX on the measures studied [27]. Changes in polyphenol content and activity may have influenced the findings over the 2‒3 d between the preparation of fresh RR tea. A study conducted in parallel with the present study, using the same batch of RR tea, demonstrated that RR tea should be prepared at least every 2 d (rather than every 2‒3 d within the present study) to minimize the potential effect of degradation of polyphenols on biological outcomes [28].

### Body composition

With respect to the effect of OVX, our study findings—higher fat mass and lower lean mass compared with SHAM, as well as higher iWAT and POAT (both of which represent visceral adipose tissue)—align with previous literature showing an increase in whole-body percent fat with a corresponding decrease in whole-body percent lean mass [[22], [23], [24], [25], [26],[32], [33], [34], [35]]. The ROI for the visceral fat mass region did not mirror the findings for whole-body fat and lean mass, though there was a trend for higher fat mass and lower lean mass in OVX groups. This is consistent with other studies using the OVX model [32,[36], [37], [38]]. Given the lack of effect seen with RR tea, it is possible that OVX promoted increased fat mass primarily through estrogen-related mechanisms rather than pathways driven by oxidative stress. However, these mechanisms are interconnected, and it is possible that multiple pathways are involved simultaneously.

The differences in body composition between surgical groups may be explained by the loss of estrogen via OVX and its impact on the differentiation of bone marrow-derived adipose tissue. This also may be reinforced by the proinflammatory state induced by estrogen withdrawal. In OVX models, estrogen supplementation has been shown to reverse the accumulation of bone marrow-derived-adipose tissue [[39], [40], [41]]. Furthermore, bone marrow-derived adipocytes and osteoblasts share a common lineage from the bone marrow stromal cells. Estrogen deficiency can suppress osteogenesis and favor adipocyte differentiation and formation [42]. This can also be further perpetuated by oxidative stress through activation of the nuclear factor-kappa B (NF-κB) pathway, which results in the secretion of proinflammatory cytokines to create an inflammation and oxidative stress-rich environment, a further stimulus for adipogenesis. This ultimately creates a positive feedback loop that promotes increased adipogenesis and decreased osteogenesis, resulting in an overall whole-body gain of fat mass and loss of lean mass [42], and this was not attenuated by RR tea supplementation.

### Metabolic outcomes

Our finding that EE did not differ between OVX and intervention groups was unexpected due to the observed higher fat mass and lower lean mass in OVX rats. A study by Witte et al. [43] compared the effects of OVX in rats for 20 d after surgery. EE was higher in OVX rats than in SHAM rats, along with increases in body mass and food intake [43]. Authors concluded that in rats, OVX weight gain is predominantly driven by hyperphagia and reduced locomotor activity [43]. Furthermore, even with pair-feeding, rats still gained weight post-OVX surgery. The authors did not have an explanation for this, given the nonsignificant differences found in EE between surgery groups [43]. However, it is important to note that this study followed the rats for 3 wk after surgery, a shorter time period than the present study. Moreover, our study demonstrated hyperphagia and increased body weight during the first several weeks post-OVX. Thus, measurement of metabolic outcomes at 12 wk post-OVX may have missed the alterations to EE or VO_2_ that may have driven the rapid body mass progression and later onset of visceral fat deposition in the OVX population. However, findings from a previous study [44] suggest that higher food intake (energy intake) observed during weeks 2 and 3 post-OVX may also be a contributor to energy imbalance. This study compared the response of lean and obese phenotype rats to OVX and reported that a higher energy intake rather than total EE was largely responsible for the energy imbalance leading to rapid weight gain within the first few weeks post-OVX [44]. Authors speculated that the contribution of energy intake and total EE following OVX may differ due to diet, housing conditions, strain, or species, and also noted that the use of *in vivo* tracers could better elucidate the mechanisms by which OVX alters metabolism [44].

### Movement data

It was expected, based on previous work that OVX would result in less cage movement [45]. A surgery × time interaction revealed a reduction in fine motor activity within the OVX group only, regardless of drink. This suggests that while at 12 wk, gross motor activity remained unchanged, fine motor activity—such as grooming or small postural movements—declined over time following OVX. This has not previously been reported within the literature—only 1 study has reported an increase in grooming in OVX rats [46]. In humans, fluctuation in fine motor skills has been associated with menstrual cycles, which may suggest a role for estrogens in fine motor control [47,48]. Potentially, a decline in fine motor activity precedes the decline in gross motor movements, or vice versa. This behavior may also be linked to cognitive health and thus should be explored further in future research. There were no other differences in movement data, including cage ambulation (all movement), and ped meters (direct locomotion), measured over the 24-h period. Similar results were found when the 12-h light or dark cycles were measured. One previous study also investigated OVX in 4-mo-old Wistar rats over a 12-wk period and performed an open field test in which a rodent is placed in the middle of a square arena divided into 25 equal boxes [32]. The number of lines crossed is indicative of exploratory/locomotive behavior. The number of horizontal and vertical movements was not significantly lower in OVX rats compared with SHAM rats [32]. Our study aligns with this finding. Given the identical endpoint timing between the studies, it can be inferred that potential OVX-induced movement changes are not detectable at 12 wk post-OVX and may not be detectable prior to this time point.

### Time budget data

Time budget data were considered, as it provides an overview of the daily time allocation of rats, allowing for detailed comparisons with baseline measurements and the SHAM groups. This can further inform whether an increase in sedentary activity, a decrease in motor activity, or a combination of both contributed to the observed changes in body lounge time and total lounge time compared with SHAM groups. Both long lounge time and total lounge time showed OVX rats trended to have longer time spent still compared with SHAM rats at endpoint (*P* = 0.065 and *P* = 0.070, respectively), whereas there were no other differences for specific time budget activities. Such results are consistent with previous work in OVX models and suggest that OVX may have influenced movement/activity and/or sedentary behavior [43,49]. The changes in movement levels may also be linked to the observed differences in fat and lean mass.

### Strengths and limitations

Key strengths of the present study include the use of the well-established OVX rat as a model of postmenopausal health and performing in-house OVX, given the rapid changes that occur within a few weeks. This allowed the RR intervention to be provided immediately after surgery. The time that lapses between surgery at the animal supplier and recovery, plus time for shipping and acclimatization to the animal facility, is often a minimum of 2 wk. This study also used *ad libitum* feeding rather than pair-feeding to mimic free living conditions and avoid psychological stress induced by pair-feeding [50]. Such psychological stress has been documented to influence overall metabolism and could lead to health issues that would be otherwise avoidable and may have influenced the results of our study [50]. Previous literature has shown that OVX results in hyperphagia for several weeks after surgery, which generally normalizes thereafter [43]. This was observed within our data and contributed to the observed body mass gain that persists after food consumption decreases. The present study is part of a larger study that also evaluated brain and bone health, including the novel object recognition test, such that it was important to minimize the psychological effects of the feeding style.

A limitation of the study includes the lack of measures of oxidative stress, antioxidant activity, or other mechanistic insights into the effect of OVX. These measures were not pursued, given the lack of effect of the RR intervention on outcomes of interest. Moreover, the sample size was not based on outcomes included in the current study. A larger sample size may have been required to detect significant differences, though the consistent finding of no effect of RR for outcomes may suggest a larger sample size may not have changed the findings from the study. Also, as previously mentioned, daily preparation of RR tea will help ensure minimal degradation of polyphenols to maximize the potential effect of the intervention [28].

### Null findings

Despite the null findings, a goal of publishing these findings is to be transparent and to thereby prevent other researchers from needlessly spending time and research funds conducting a study with the same or similar study objective. These null findings can guide other researchers in modifying experimental designs if wanting to test a hypothesis about RR intervention on health outcomes. Also, it could be useful to repeat a RR group within a future design to confirm the null finding and to ensure reproducibility of findings from the present study.

In conclusion, 12 wk of RR tea consumption had no effect on body composition, metabolic outcomes, or movement in an OVX rat model. This study adds a comprehensive dataset to the literature on the effects of OVX on a 6-mo-old female rat model and is the first to document the effects of a RR tea intervention in the OVX rat model. The contrasting findings of the present study with those of previous polyphenol interventions (particularly the effect of providing green tea) may prove useful to help identify specific chemical structures within specific polyphenols or other bioactives that may or may not mediate potential effects on body composition and other metabolic outcomes studied. This would provide a structure-function basis for selecting and studying future bioactives using this model.

## Author contributions

The authors’ responsibilities were as follows – RSF, MKG, SM, DBK, REKM, WEW: designed research; RSF, MAI, MKG, SM, DBK: conducted research; RSF: analyzed data; RSF, MAI, REKM, BDR, WEW: wrote the paper; RSF, REKM, WEW: had primary responsibility for final content; and all authors: read and approved the final manuscript.

## Data availability

The study data are openly available in the Nutrition, Bone, and Oral Health Research Group (WEW) Dataverse: https://doi.org/10.5683/SP3/NSSXDZ.

## Funding

This research was supported by an Natural Sciences and Engineering Research Council (NSERC) of Canada Discovery Grant to WEW. WEW holds a Senior Research Fellowship from the Faculty of Applied Health Sciences at Brock University. MAI received student funding through the Ontario Graduate Scholarship Program.

## Conflict of interest

The authors report no conflicts of interest.
