# Supplementary material for: Effect of Supplementation with Red Rooibos Tea on Body Composition, Metabolic Outcomes, and Movement in Ovariectomized Sprague-Dawley Rats
Source: Curr Dev Nutr. 2026 Feb 6;10(3):107656. doi: 10.1016/j.cdnut.2026.107656 (PMC12955642; doi:10.1016/j.cdnut.2026.107656)
Supplement: Multimedia component 1 [file mmc1.docx]

**Effect of supplementation with red rooibos tea on body composition, metabolic outcomes and movement in ovariectomized Sprague-Dawley rats.**

**Rebekah S. Feld**

**
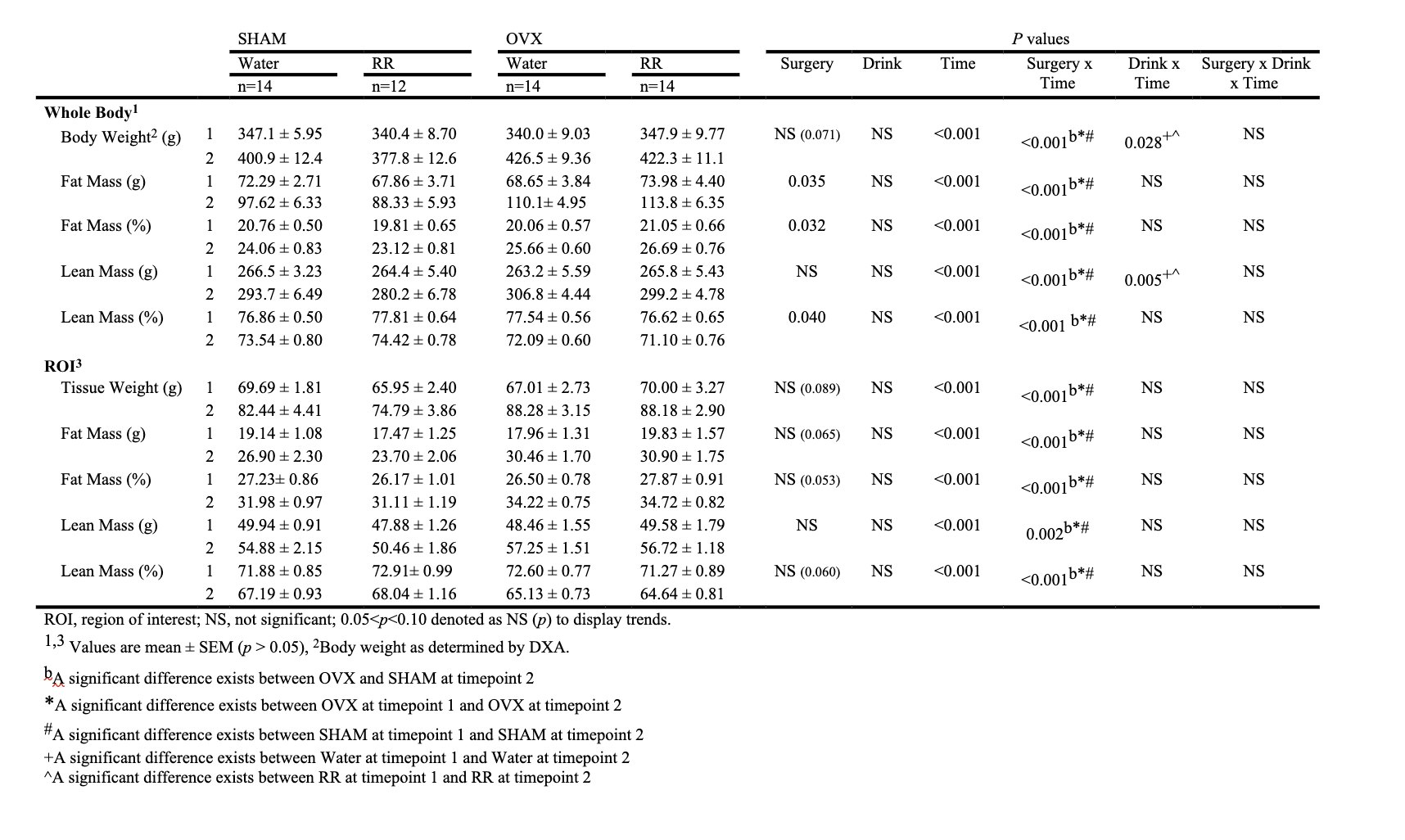
Supplementary Table 1**: Fat and lean mass for whole body and the visceral ROI
